# Psycho-Oncological Intervention Through Counseling in Patients With Differentiated Thyroid Cancer in Treatment With Radioiodine (COUNTHY, NCT05054634): A Non-randomized Controlled Study

**DOI:** 10.3389/fpsyg.2022.767093

**Published:** 2022-02-25

**Authors:** Nuria Javaloyes, Aurora Crespo, M. Carmen Redal, Antonio Brugarolas, Lara Botella, Vanesa Escudero-Ortiz, Manuel Sureda

**Affiliations:** ^1^Plataforma de Oncología, Hospital Quirónsalud Torrevieja, Torrevieja, Spain; ^2^Servicio de Medicina Nuclear, Hospital Universitari Sant Joan, Alicante, Spain; ^3^Servicio de Medicina Nuclear, Hospital Clínico Universitario de Valencia, Valencia, Spain; ^4^Facultad de Ciencias de la Salud, Grupo de Investigación en Farmacia y Nutrición Clínica, Universidad CEU Cardenal, Elche, Spain

**Keywords:** cancer, oncology, psycho-oncology, psychotherapy, thyroid cancer, radioisotope therapy, quality of life

## Abstract

**Background:**

Diagnosis and treatment of differentiated thyroid carcinomas (DTC) cause anxiety and depression. Additionally, these patients suffer hormonal alterations that are associated with psychological symptoms (e.g., changes in mood, emotional instability, and memory loss). This study aims to evaluate the effectiveness of a psycho-oncological intervention based on counseling to reduce anxiety and depression related to the treatment in patients with DTC.

**Methods:**

A non-randomized controlled study, with two groups [experimental group (EG), *n* = 37, and control group (CG), *n* = 38] and baseline and posttreatment measures, was designed. Patients in the EG received a psycho-oncological intervention based on counseling in addition to the standard treatment. The independent variable was the assigned group and the dependent one was the evolution of anxiety and depression, which were analyzed separately, and both were evaluated using the Hospital Anxiety and Depression Scale. Other relevant covariables related to the quality of life (QoL) were also analyzed using Short Form-36 Health Survey and Psychological General Wellbeing Index scales.

**Results:**

The difference of the posttreatment-baseline variation showed a statistically significant reduction in anxiety and depression in the EG in relation to the CG (*p* < 0.001). The mean of the Psychological General Wellbeing Index scales score increased significantly in the EG (*p* < 0.001) and decreased significantly in the CG (*p* < 0.001). All the baseline and the posttreatment scores of the variables evaluated showed a statistically significant improvement in the EG vs. the CG.

**Conclusion:**

This study demonstrates significant benefits of psycho-oncological intervention based on counseling in anxiety, depression, QoL, and wellbeing of the patient with differentiated thyroid carcinomas.

## Introduction

Thyroid cancer (TC) is the most frequent cancer of the endocrine system and the main cause of death due to tumors originating in this system. Differentiated thyroid carcinomas (DTC) originate from follicular cells and constitute up to 90% of all TC.

The primary treatment for DTC is surgery (Soriano et al., 2012). Only DTC are susceptible to being treated with radioactive iodine therapy (RIT) because the tumor cell maintains the ability to capture iodine. RIT is administered orally and is absorbed selectively by any remaining thyroid tissue, including TC cells elsewhere in the body, without harming normal tissues (Luster et al., 2008; Soriano et al., 2012).

Cancer diagnosis and the associated treatment usually cause a deep emotional wound to the patient and family. TC surgery affects the anterior portion of the neck, one of the most visible areas of the organism. RIT constitutes an additional source of stress. Hormonal alterations due to the treatment itself or to the controls needed for follow-up can cause changes in mood, lack of concentration, emotional instability, and/or memory loss among others (Cooper et al., 2006; Saravanan et al., 2006).

The goals of psycho-oncological interventions are to help patients to better come to terms with their treatment, to decrease feelings of alienation, isolation, helplessness, and hopelessness, to reduce anxiety related to treatments, and to clarify perceptions and false information (Arranz and Cancio, 2014).

Reviewing the literature, it is observed that, despite methodological deficiencies and contradictory results present in some publications, the psychological interventions usually improve the adaptation of oncological patients to the disease process. Some of the primary work generating specific treatment programs were based on the transactional theory of stress and coping developed by Lazarus and Folkman (1987) or the cognitive psychology and cognitive behavioral therapy (CBT) developed by Beck (Moorey et al., 1993; Fawzy et al., 1995).

Counseling (Espinoza, 2014), CBT (Trask et al., 2003), individual psychoeducational interventions (Lapid et al., 2007), and short-term group therapy (Naaman et al., 2009) have shown relevant efficacy to improve and promote quality of life (QoL). Counseling (van der Meulen et al., 2013), individual psychoeducational interventions (Lee et al., 2014), relaxation (Dolbeault et al., 2009), CBT (Moorey et al., 1998; Kissane et al., 2003; Narváez et al., 2008), group therapy (Cameron et al., 2007), and mindfulness (Bränström et al., 2012) have shown positive effects with statistically significant improvements in the state of mind. Counseling also significantly improves the psychological adaptation, pain management, and sexual functioning in mastectomized women (Maguire et al., 1983; Jahn et al., 2014). In addition, it has been shown that interventions based on counseling make the cancer patient feel better treated, more satisfied, and more motivated to maintain healthy lifestyles (Hersch et al., 2009; Galway et al., 2012; Naumann et al., 2012).

In the case of DCT, there are studies of alterations in QoL and mood (Tagay et al., 2005; Tagay et al., 2006; Aschebrook-Kilfoy et al., 2015; Buchmann et al., 2015; Badihian et al., 2016), but the research on the effectiveness of psycho-oncological interventions directed to correct them (search in PubMed with keywords “thyroid cancer” and “psychological intervention” in March 2020) is scarce. Wu et al. (2016) explored the effect of behavioral intervention and counseling on QoL and mental health in patients with DTC treated with surgery and RIT. They concluded that 1 year of behavioral and psychological intervention in the experimental group (EG) patients, performed by specifically trained and qualified nurses, improved overall QoL and significantly reduced the symptoms of depression and anxiety (*p* < 0.05) (Wu et al., 2016).

Research on the evaluation of the state of mind, QoL, and wellbeing of patients with DTC treated with RIT and research on the efficacy of psycho-oncological interventions to improve these parameters contribute to increase empirical knowledge in psycho-oncology, optimizing the treatment protocols for patients with DTC, and comprehensive care for their psychological and emotional needs. At present, despite the acknowledged need for emotional support for cancer patients, the effective integration of psycho-oncologists in the multidisciplinary team (MDT) in oncology is far from common (Singer et al., 2016).

COUNTHY consisted of a prospective pilot study, with EG and control (CG) groups and baseline and posttreatment measures. Patients in EG received a psycho-oncological intervention based on counseling (PIBC) following the scheme proposed by Arranz and Cancio (Arranz and Cancio, 2014) in addition to the standard treatment. The evolution of anxiety and depression was evaluated using the Hospital Anxiety and Depression Scale (HADS) (Zigmond and Snaith, 1983; Herrmann, 1997). The aim of the study was to evaluate the effectiveness of a PIBC in reducing anxiety and depression in patients with DTC. Other QoL relevant items were also evaluated using the Short Form-36 Health Survey (SF-36) (Molina, 2005; Vilagut et al., 2005) and Psychological General Wellbeing Index (PGWBI) scales (Martin and Ware, 2004).

## Patients, Materials, and Methods

Patients diagnosed with DTC, treated with thyroidectomy and candidates to RIT to be treated in Nuclear Medicine (NM) Service of Hospital Quirónsalud Torrevieja, were considered to be included in the COUNTHY study.

The inclusion criteria were as follows: diagnosis of DTC, initial treatment by total thyroidectomy, treatment with RIT after physiological stimulation of thyroid-stimulating hormone (TSH) by suppression of hormone replacement therapy (HRT) for a month, fluid Spanish speaker, 18 years or above, and obtaining informed consent. The exclusion criteria were as follows: diagnosis or treatment for psychiatric disorders, any type of illness with serious impact on medical condition, and inability to communicate or to process the information.

Those patients who met the inclusion criteria were individually informed about the existence of the study. If they accepted inclusion, they were alternatively assigned to the EG or CG, according to their order of entry. Accrual continued until the calculated sample size was complete.

A non-randomized controlled study with baseline and posttreatment measures was designed to assess the effect of PIBC in reducing both dependent variables, namely, anxiety and depression. The effect of therapy in each patient was assessed with the change in anxiety and depression at the end of the follow-up.

The sample size was calculated for two independent samples for both anxiety and depression, considering a minimum goal of a 3-point reduction in the HADS. This value was extracted from the data previously published for patients with DTC after surgery and treated with HRT [baseline SD at 3.7 points (anxiety) or 3.4 (depression) and the SD of change posttreatment at 3.6 points (anxiety) or 3.7 (depression)] (Tagay et al., 2005). The relationship between EG and CG is 1:1, and a bilateral α risk of 5% and a β risk of 10% are accepted. Along with the duration of the study, 10% of possible losses are expected; therefore, it will be necessary to recruit a minimum of 32/0.90 = 35.5 (36) subjects per group, in total 36 × 2 = 72 patients.

Patients allocated in the CG were treated according to the standard protocol of the NM Service while those in the EG received 4 sessions of individual PIBC distributed during the treatment, in addition to the standard protocol (refer to Table 1). PIBC sessions followed the scheme proposed by Arranz and Cancio (2014) (refer to Supplementary Annex 1) and were administered by 2 psycho-oncologists, with the specific assignation of each patient to one of them. The duration of each session was 45 min.

**TABLE 1:** Intervention itinerary for control group (CG) and experimental group (EG) patients.

	Control group		Experimental group	
Day 0	Baseline interview according to protocol	Nuclear Medicine Specialist	Baseline interview according to protocol	Nuclear medicine specialist
	● Sociodemographic data collection ● Pre-intervention evaluation	Nuclear Medicine specialist	● Sociodemographic data collection ● Pre-intervention evaluation ● PIBC	**Psycho-oncologist**
3rd week			● PIBC	**Psycho-oncologist**
4th week	Admission for treatment	Nuclear Medicine Specialist	Admission for treatment	Nuclear Medicine Specialist
5th week	Post-treatment image study	Nuclear Medicine Specialist	Post-treatment image study ● PIBC	Nuclear Medicine Specialist + **Psycho-oncologist**
8th week	● Post-intervention evaluation	Nuclear Medicine Specialist	● Post-intervention evaluation ● PIBC	Nuclear Medicine Specialist + **Psycho-oncologist**

Once completed the period of suppression of HRT, RIT was administered in a radio-protected and isolated room where the patient remained for usually 2–3 days. On discharge, HRT was reintroduced.

### Psychological Evaluation Instruments

Anxiety and depression were evaluated through the HADS (Zigmond and Snaith, 1983; Herrmann, 1997). The other relevant covariables related to QoL as mood, perceived wellbeing, and emotional impact were evaluated through the SF-36 questionnaire and the PGWBI of Dupuy (Martin and Ware, 2004; Molina, 2005; Vilagut et al., 2005).

After the first interview with the NM specialist, only the patients assigned to the EG received a PIBC session adapted to their individual needs (day 0). Three weeks later, in HRT withdrawal, EG patients received the second session of PIBC.

During the week after hospital discharge from RIT, the patients had to comply at home with the measures to avoid irradiation to third parties. All patients were interviewed at the end by the NM specialist and the EG ones additionally by the psycho-oncologist, who conducted a third PIBC session.

The last session of the study, 4 weeks after RIT and 8 weeks from the beginning, was carried out with all the patients that received the entire battery of tests (HADS, SF-36, and PGWBI). The EG ones also had the final PIBC session with the psycho-oncologist (see Table 1).

The independent variable was the allocation to the EG or CG. The main dependent variables were anxiety and depression, independently considered and evaluated. Other covariables related to QoL were complementarily evaluated, also with baseline and posttreatment measures.

Effect size measure was calculated with Cohen’s *d* for the difference between EG and CG in the primary outcome variable at posttreatment, for both anxiety and depression. A *post hoc* calculated statistical power analysis (power = 1-β) was performed using a two-sample means test with a bilateral test and a significance level of 5% (α = 0.05).

For the analytical study of the data obtained, the association between categorical variables of the study, both in the CG and in the EG, was performed using Pearson’s chi-square test or using Fisher’s exact test in those cases with less than 5 expected observations. The difference between the baseline and posttreatment measure of quantitative variables was evaluated, in each group, through a study of the difference of the means for paired data through the Student’s *t*-test (for paired samples). The difference of the means of the quantitative variables between the different groups of the study was carried out with the Student’s *t*-test for independent samples if the variable presented a normal distribution in its values, or by the Mann-Whitney *U*-test if the variable presented a non-normal distribution. All hypothesis tests were carried out through a bilateral test, with a significance level of α = 0.05. In addition, an analysis of covariance (ANCOVA) was carried out, with the posttreatment scores of the outcome variable as the dependent variable, the intervention condition as the independent variable, and the baseline score of the outcome variable as the covariate, thus adjusting for baseline imbalances of the outcome variable.

The statistical analysis was carried out using the SPSS19.0 program (IBM, Armonk, NY, United States).

COUNTHY study protocol obtained the approval of the Committee of Ethics and Clinical Trials of the Hospital Quirónsalud Torrevieja (act ref 20130116-1) and was registered in ClinicalTrials.gov (NCT05054634). This study was performed in accordance with Good Clinical Practice standards and the Declaration of Helsinki.

## Results

Between June 2013 and September 2014, a total of 75 patients were included in the study, 37 in the EG and 38 in the CG. No patient declined to be included. No statistically significant differences were observed in sociodemographic characteristics between both groups (Table 2).

**TABLE 2:** Sociodemographic characteristics of patients.

	Control group (*N* = 38)		Experimental group (*N* = 37)		
Variable	N	%	N	%	*p*
**Sex**					
Men	12	31.6	9	24.3	0.330^a^
Women	26	68.4	28	75.7	
**Studies level**					
No studies	5	13.2	1	2.7	
Primary	13	34.2	20	54.1	0.187^b^
Secondary	13	34.2	9	24.3	
University	7	18.4	7	18.9	
**Cancer family history**					
No	16	42.1	10	27.0	0.129^a^
Yes	22	57.9	27	73.0	
**Poor prognostic factor**					
No	16	42.1	17	46.0	0.459^a^
Yes	22	57.9	20	54.1	
**Previous treatment**					
No	30	79.0	28	75.7	0.475^a^
Yes	8	21.1	9	24.3	
**Histological type**					
Follicular	6	15.8	3	8.1	0.253^b^
Papillary	32	84.2	34	91.9	

*^a^Pearson’s chi-square test.*

*^b^Fisher’s exact test.*

During the baseline evaluation, 21/37 patients in the EG (57%) did not show symptoms of anxiety, while in the posttreatment evaluation after PIBC, the number increased to 35/37 (95%). In the CG half of the patients in both, the baseline and the posttreatment evaluation did not have anxiety symptoms. However, in the posttreatment evaluation after the RIT, clinical cases of anxiety increased from 4/38 (11%) to 9/38 (24%).

After PIBC, the subset of patients without depression symptoms in the EG increased from 33/37 (89%) to 36/37 (97%). In the CG, 31/38 (82%) patients did not have symptoms of depression in the baseline evaluation, whereas, after RIT, there were 25/38 (66%) patients. Calculating the effect size, the Cohen’s *d* for the difference between baseline and posttreatment in EG in the primary outcome variable anxiety was *d* = 1.32, whereas it was *d* = −0.21 in the CG. For the primary outcome variable depression, the effect size obtained was *d* = 0.90 in EG and *d* = −0.50 in CG.

The *post hoc* calculated power analysis for posttreatment anxiety in both EG and CG was 100%. The same result was obtained for posttreatment depression in EG and CG.

Analysis of means comparison (Table 3) shows a decrease in the mean scores after the PIBC in both anxiety and depression, and this decrease was statistically significant in both cases (*p* < 0.001). In the CG, the mean score of anxiety showed a non-statistically significant increase after the RIT (*p* = 0.162), but the mean score of depression after RIT showed a statistically significant increase (*p* = 0.018).

**TABLE 3:** Comparison of the differences (posttreatment-baseline) between the CG and EG.

Variable HADS	Control group				Experimental group			
	Pre	Post	Mean difference		Pre	Post	Mean difference	
			d (SE) (CI-95%)	*p*			d (SE) (CI-95%)	*p*
Anxiety	7.0	7.7	0.8 (0.8) (–0.8/2.4)	0.1730	7.1	3.1	–4.0^§^ (0.7) (–5.4/–2.6)	**<0.001^±^**
Depression	4.5	6.1	1.6 (0.7) (0.1/3.0)	**0.0160**	4.4	2.2	–2.2 (0.6) (–1.1/–3.4)	**0.0001**

*^§^ Normal distribution. The rest of the distributions are not normal (Shapiro-Wilk test). Contrast of hypotheses (p) using the Mann-Whitney U-test for all comparisons, except ^±^using Student’s t-test for independent samples. Statistically significant differences are highlighted in bold.*

An ANCOVA between groups, unifactorial, was applied with two treatment conditions (EG and CG) as an independent variable, to observe its effect on posttreatment anxiety as a dependent variable. Pretreatment anxiety scores were used as a covariate. The data obtained from the regression model show that the group factor (EG or CG) explains 56.5% of the variation in the score of the posttreatment anxiety.

The results indicate that patients in the CG present higher posttreatment anxiety scores [mean (SD) = 7.74 (3.72)] than those in the EG [3.05 (2.33)], a statistically significant difference [*F*(1, 72) = 61.7, *p* < 0.001].

The same ANCOVA between groups, unifactorial, with two treatment conditions (EG and CG) as an independent variable, was carried out in the case of depression to observe the effect on the posttreatment depression as a dependent variable. Pretreatment depression scores were used as a covariate. The data obtained from the regression model show that the group factor (EG or CG) explains 40.1% of the variation in the score of the posttreatment depression.

The results indicated that the patients in the CG presented higher postdepression scores [mean (SD) = 6.05 (2.98)] than those in the EG [2.16 (1.92)], a statistically significant difference [*F*(1, 72) = 46.1, *p* < 0.001].

In the EG, both the symptomatology of anxiety (baseline-posttreatment difference = −4) as of depression (baseline-posttreatment difference = −2) improved significantly (*p* < 0.001), but in the CG, the symptoms of anxiety worsened (baseline-posttreatment difference = 1) as of depression (baseline-posttreatment difference = 2), although only these latter were statistically significant (*p* < 0.001) (Table 3).

A statistically significant increase was observed in the mean scores of all the variables of the SF-36 after the PIBC in the EG (*p* < 0.001). In the CG, there was a generalized decrease in the mean of the scores of all the variables evaluated after the RIT, although this decrease was statistically significant only in 4 of them as follows: Physical performance (*p* = 0.003), social function (*p* = 0.009), vitality (*p* = 0.030), and mental health (*p* = 0.003) (Table 4).

**TABLE 4:** Comparative results (posttreatment-baseline) of the dimensions of the SF-36 and PGWBI in the CG and EG.

	Baseline	Post-treatment	Difference				
	Mean	Mean	Mean	IC-95%			*p* *
**Control group (*N* = 38)**							
Health change over time	46.6	41.7	–4.9	–11.2	–	1.5	0.128
General health	55.7	49.5	–6.1	–12.5	–	0.1	0.053
Physical function	77.2	74.9	–2.4	–11.2	–	6.5	0.592
Physical performance	66.9	47.8	–19.0	–31.4	–	–6.7	**0.003**
Emotional performance	68.2	63.3	–4.9	–16.5	–	6.6	0.393
Social function	74.0	59.6	–14.5	–25.2	–	–3.8	**0.009**
Physical pain	78.3	73.8	–4.5	–12.1	–	3.2	0.244
Vitality	58.5	50.0	–8.5	–16.1	–	–0.9	**0.030**
Mental health	63.7	53.9	–9.8	–16.1	–	–3.5	**0.003**
PGWBI	67.1	58.9	–8.1	–14.9	–	–1.4	**0.009**
**Experimental group (*N* = 37)**							
Health change over time	32.4	56.1	23.6	17.2	–	30.1	**<0.001**
General health	54.1	76.7	22.6	16.0	–	29.3	**<0.001**
Physical function	76.8	95.1	18.4	9.6	–	27.1	**<0.001**
Physical performance	40.0	81.0	40.9	27.0	–	54.8	**<0.001**
Emotional performance	73.9	94.7	20.8	8.1	–	33.4	**<0.001**
Social function	75.0	87.5	12.5	7.0	–	17.9	**<0.001**
Physical pain	81.7	91.2	9.6	1.2	–	17.9	**<0.001**
Vitality	57.5	79.5	22.0	13.9	–	30.1	**<0.001**
Mental health	68.2	81.9	13.7	9.0	–	18.4	**<0.001**
PGWBI	67.5	83.8	16.3	11.6	–	21.0	**<0.001**

*Student’s t-test for paired data. Statistically significant differences are highlighted in bold.

In the evaluation post-RIT, analyzing the PGWBI data in the EG, the results showed a statistically significant improvement (baseline-posttreatment difference = 16, *p* < 0.001) changing from the range of moderate baseline discomfort to the range of positive wellbeing (range: 73–110), with a final average of 84 points. Meanwhile, in the CG, there was a decrease in perceived welfare (baseline-posttreatment difference = −8), going from the range of baseline moderate discomfort to the range of severe discomfort with an average score of 59 (range: 0–60), statistically significant (*p* < 0.001).

## Discussion

This study has evaluated the effectiveness of the PIBC on anxiety and depression in patients with DTC after RIT using a prospective design with EG and CG. Other relevant parameters influencing QoL and general psychological performance were also evaluated.

In the baseline evaluation, half of the patients in both groups did not present symptoms of anxiety and more than 80% did not present depressive symptoms. The average score of the PGWBI was 67 points in both groups, indicating a moderate baseline discomfort in the total sample of patients (defined by a score in the range of 61–72).

Treatment of choice for DTC is combined ablation of the thyroid (surgery + RIT). The benefits from this combination include lower recurrence rates and higher survival and higher cure rates (Cooper et al., 2009). However, the elements of the combination may cause emotional impacts in the patient despite increased therapeutic efficiency. It should be noted that the baseline evaluation in this study was carried out on all patients after surgery, before the hypothyroidism secondary to withdrawal of HRT and prior to the RIT, while the posttreatment evaluation was carried out on all patients post-RIT, having received those of the EG 4 sessions of PIBC in addition to the standard treatment. In this study, surgery does not affect significantly QoL, in coincidence with some results of the literature (Dagan et al., 2004; Sung et al., 2011). However, others reported that the surgery does affect the QoL finding a positive and directly proportional correlation between the time after thyroidectomy, the degree of psychological wellbeing, and the QoL of patients with DTC (Novoa et al., 2010). Alternative procedures of thyroidectomy from Korea or China have been recently published, trying to avoid the undesirable effect of a scar on the neck through axillary access with robotic surgery (Lee and Chung, 2013; Lee et al., 2013; Liu and Ng, 2016).

Due to the worsening in all dimensions in the CG in this study, it can be stated that the factors that have mainly affected the emotional state of patients with DTC are those that surround the RIT, as previously reported (Dagan et al., 2004). Dagan et al. (2004) evaluated the QoL of 78 patients with DTC and concluded that the greatest degree of emotional distress occurred during HRT withdrawal. Botella-Carretero et al. (2003) studied the psychological performance and the QoL of patients with DTC in a situation of hormone suppression and concluded that both variables are affected by the withdrawal of HRT both before the RIT and in the follow-up of the disease.

The design of this study in two groups, namely, EG and CG, is justified for favoring a more rigorous analysis of the possible differences between intervention and non-intervention. Instead of assuming the null hypothesis that with no intervention there are no differences, the difference between the baseline (after surgery) and the posttreatment (post-RIT) variation was analyzed in each group and for each variable. The comparison between the baseline and posttreatment variation in both groups showed a reduction in the anxiety in the EG of −5 points in relation to the CG, and a reduction of the depression in the EG of −4 points compared with the CG (Table 3). PIBC significantly improved the mood (anxiety, depression) of the EG patients compared with the CG (*p* < 0.001).

The SF-36 dimension that worsened less in the CG after the RIT was the physical function, associated with the performance of activities such as walking, running, lifting or moving heavy objects, bending over, kneeling, squatting, bathing, or dressing. When the final evaluation was performed, 4 weeks after discharge from the RIT, the daily life activities would not be normalized yet, so the parameters that measure this dimension were not fully present at the moment of the evaluation. It would be interesting to prospectively evaluate the possible benefit of adding a new measurement to the protocol when patients have already fully incorporated these activities.

Physical performance was the dimension that got worse in the CG (difference between baseline and posttreatment average = −19), the one with higher improvement after PIBC in the EG (difference between baseline and posttreatment average = 41), and the one with the greatest difference in the EG compared with the CG (60 points) (Table 4). This dimension evaluates the limitation or difficulty to perform some kind of activity and the perception of doing fewer activities than the patient would have wanted to do. In PIBC sessions, adaptation capacity was addressed, strengthening existing resources already inside the patient and/or training in new resources to facilitate adaptation to the situation. The results could be interpreted in the sense that PIBC facilitated self-perception in a less restrictive way, adapting better to the new situation and getting better scores in the physical performance dimension of the SF-36. Wu et al. (2016) explored the effect of behavioral intervention and counseling on QoL and mental health in patients with DTC treated with surgery and RIT. They found a statistically significant improvement in QoL, depression, and anxiety, in line with those described in this study (Wu et al., 2016). The lack of additional studies in this field, together with the data reported in this study, paves the way for a significant improvement in the MDT of the patient with DTC, also strengthening previous evidence about the benefits of the inclusion of the psycho-oncologist in the team (Zigmond and Snaith, 1983; Buchmann et al., 2015; Duan et al., 2015).

### Clinical Implications

Treatment of DTC causes relevant emotional impacts in the patient. This study reports a significant benefit in anxiety and depression when a psycho-oncological intervention based on counseling as described is added to the standard treatment.

A concrete and detailed definition of the psycho-oncological intervention based on counseling employed is fundamental to permit the reproducibility of experimental studies, thus helping to achieve the empirical evidence necessary for advancement in psycho-oncology research (Supplementary Annex 1). This study makes a significant contribution in this line.

### Study Limitations

Single-center characteristics, the non-randomized design, and the limited sample size constitute the major limitations of this pilot study. Additionally, social and family support constitutes a known protective factor of emotional impact in cancer patients (Rodríguez-Marín et al., 1993). In this study, this factor has not been considered in the baseline evaluation although it has been evaluated and intervened in the PIBC sessions with the EG. Taking all these factors into consideration, the encouraging findings presented have to be confirmed in further larger, randomized, and when possible, multicentric trials.

## Conclusion

Patients with DTC present no significant alterations in anxiety and depression, before RIT, with significant worsening observed after RIT. PIBC significantly improves anxiety and depression and can contribute to improve other dimensions related to QoL. According to these findings, the psycho-oncologist must be part of the integral MDT for the treatment of DTC and probably of all the MDT in oncology. Further studies have to confirm these encouraging results.

## Data Availability Statement

The raw data supporting the conclusions of this article will be made available by the authors, without undue reservation.

## Ethics Statement

The studies involving human participants were reviewed and approved by the Committee of Ethics and Clinical Trials of the Hospital Quirónsalud Torrevieja (act ref 20130116-1). The patients/participants provided their written informed consent to participate in this study.

## Author Contributions

NJ obtained experimental data and wrote the manuscript. AC, MR, and LB obtained experimental data and reviewed the manuscript. AB contributed to the experimental design and analysis of experimental data. VE-O collaborated with the analysis of experimental data and writing and revision of the manuscript. MS contributed to the design and wrote the manuscript. All authors contributed to the article and approved the submitted version.

## Conflict of Interest

The authors declare that the research was conducted in the absence of any commercial or financial relationships that could be construed as a potential conflict of interest.

## Publisher’s Note

All claims expressed in this article are solely those of the authors and do not necessarily represent those of their affiliated organizations, or those of the publisher, the editors and the reviewers. Any product that may be evaluated in this article, or claim that may be made by its manufacturer, is not guaranteed or endorsed by the publisher.

## Abbreviations

CBT: Cognitive Behavioral Therapy
CG: Control Group
DTC: Differentiated Thyroid Carcinoma
EG: Experimental Group
HADS: Hospital Anxiety and Depression Scale
HRT: Hormone Replacement Therapy
NM: Nuclear Medicine
PIBC: Psycho-oncological Intervention Based on Counseling
PGWI: Psychological General Wellbeing Index
QoL: Quality of Life
RIT: Radioiodine Therapy
SF-36: Short Form-36 Health Survey
TC: Thyroid Carcinoma
TSH: Thyroid Stimulating Hormone.
