# Supplementary material for: Psycho-Oncological Intervention Through Counseling in Patients With Differentiated Thyroid Cancer in Treatment With Radioiodine (COUNTHY, NCT05054634): A Non-randomized Controlled Study
Source: Front Psychol. 2022 Feb 25;13:767093. doi: 10.3389/fpsyg.2022.767093 (PMC8914112; doi:10.3389/fpsyg.2022.767093)
Supplement: Supplementary file 1 [file Table_1.DOCX]

Annex 1. Schema of PIBC sessions.

Each session was based step by step on the following guide, proposed by Arranz and Cancio^5^:

1. To identify age, family situation and/or concomitant diseases.

2. To identify concerns, situations of fear and needs in the most specific way possible. To facilitate emotional expression.

3. To help to hierarchize what has been identified, empathizing with their emotions and the underlying values ​​of what is expressed.

4. To identify resources and capacities, whether internal or external, to facilitate adaptation.

5. To provide information. To identify what the patient knows, what who wants to know and what he/she has understood about what worries him/her.

6. To address concerns, combining them with the resources and capabilities available to the person and with others that can be suggested. To listen to what the patient and his/her non-verbal communication.

7. To clarify the different options evaluating pros and cons.

8. To assist in decision-making based on congruence with the patient's own values ​​and resources.

9. To summarize and to plan the future.
